# Physiological and Biochemical Responses in Two Ornamental Shrubs to Drought Stress

**DOI:** 10.3389/fpls.2016.00645

**Published:** 2016-05-12

**Authors:** Stefania Toscano, Elisa Farieri, Antonio Ferrante, Daniela Romano

**Affiliations:** ^1^Department of Agriculture, Food and Environment, University of CataniaCatania, Italy; ^2^Department of Agricultural and Environmental Sciences – Production, Landscape, Agroenergy, Università degli Studi di MilanoMilano, Italy

**Keywords:** antioxidant enzymes, *Eugenia uniflora* L., gas exchange, lipid peroxidation, *Photinia × fraseri* Dress, water deficit

## Abstract

Drought stress is one of the most important abiotic stress limiting the plant survival and growth in the Mediterranean environment. In this work, two species typically grown in Mediterranean areas with different drought responses were used. Two shrubs, with slow (*Photinia × fraseri* Dress ‘Red Robin’) or fast (*Eugenia uniflora* L. ‘Etna Fire’) adaptation ability to drought, were subjected to three water regimes: well-watered (WW), moderate (MD), and severe (SD) drought stress conditions for 30 days. Net photosynthetic rate, stomatal conductance, maximum quantum efficiency of PSII photochemistry (Fv/Fm), relative water content (RWC), chlorophyll content, proline, malondialdehyde (MDA), and antioxidant enzyme activities (superoxide dismutase, catalase, and peroxidase) were measured. Results showed that RWC and proline were higher in *Eugenia* than in *Photinia*, demonstrating the greater tolerance of the latter to the water stress. The drought stress levels applied did not compromise photosynthetic efficiency through stomatal regulation, while a reduction of Fv/Fm ratio was observed at the end of the experimental period. MDA significantly increased after 30 days in both species. The antioxidant enzyme activities showed different responses to water stress conditions. In both species, the water stress scores showed positive, while proline content showed negative correlations with all physiological parameters.

## Introduction

Water stress, in combination with high temperatures and high levels of irradiance, is considered one of the most severe environmental stress that hampers plant survival and productivity in arid and semi-arid areas (Morales et al., 2013). The climate of Mediterranean region is characterized by high temperature, high vapor pressure deficit, high radiation levels, and low rainfall, during the vegetation seasons. These conditions lead to negative consequences on the plants growth under stress conditions. Plants under drought stress showed a series of morphological, physiological, biochemical, and molecular changes that adversely affect plant growth and productivity (Wang et al., 2001). Plants under drought conditions decrease net photosynthesis rate and transpiration; these physiological responses are common in zones where the evaporative demand is very high (Feng and Cao, 2005). Protection mechanisms against excess reducing power are thus an important strategy under water stress (Chaves et al., 2009). When photosynthesis is reduced and light excitation energy is in excess of that used in photosynthesis, over-excitation of the photosynthetic pigments in the antenna can occur, leading to the accumulation of reactive oxygen species (ROS) in chloroplasts (Munné-Bosch et al., 2003). The ROS such as O_2_, H_2_O_2_ and OH⋅ radicals can directly damage the phospholipids of the cell membrane and increase lipid peroxidation (Mittler, 2002). Water stress induces the overproduction of ROS and consequently increase the lipid peroxidation membranes measured as MDA content, which is the final product of lipid peroxidation and it is a well-known marker of oxidative damage (Moller et al., 2007). In the case of signs and/or oxidative damage, plants put in place strategies to balance the ROS production and the antioxidant enzyme activities (Moller et al., 2007). To minimize the effects of oxidative stress, plants have developed a complex enzymatic and non-enzymatic systems, such as low molecular weight antioxidants (glutathione, ascorbic acid, carotenoids) and ROS scavenging enzymes (SOD, GPX, CAT, APX; Apel and Hirt, 2004). Several studies reported that the species subjected to mild and/or moderate water stress conditions increased the activity of antioxidant enzymes (Ge et al., 2014), such as SOD and GPX. The most important antioxidant enzymes are SOD (EC 1.15.1.1), CAT (EC 1.11.1.6), and GPX (EC 1.11.1.7). SOD converts O_2_^-^ into H_2_O_2_ and O_2_, and CAT and GPX scavenge H_2_O_2_ into H_2_O (Reddy et al., 2004). Some authors did not observe water stress effects on the enzyme activities (Delfine et al., 2001), while others observed a significant increase of RuBisCo oxygenase activity in plants subjected to drought (Maroco et al., 2002). These differences are related to the different environmental conditions, species and drought levels of the study that were carried out (Bota et al., 2004). Previous studies indicated that higher activity levels of antioxidant enzymes can contribute to better drought tolerance by increasing the protection capacity against oxidative damage (Türkan et al., 2005). Many species subjected to abiotic stress conditions enhance the antioxidant enzyme activities, which are connected to higher proline content (Ashraf and Foolad, 2007). Proline is one of the most important cell solutes and its high concentration is considered an indicator of tolerance to water stress (Liu et al., 2014). Proline at high concentrations may protect plants from environmental stresses through its contribution to cellular osmotic adjustment, detoxification of ROS, protection of membrane integrity, and stabilization of enzymes/proteins (Ashraf, 2009). Water shortage reduces plant growth especially in the Mediterranean areas, therefore, the research has been focused on the response of native shrub species to water stress (Munné-Bosch and Peñuelas, 2004), while less extensive analyses are performed against exotic species, which are widely used as ornamental plants (Álvarez et al., 2011). *Photinia × fraseri* ‘Red Robin,’ evergreen shrub, is a popular ornamental plant due to its strikingly red young leaves (Deng et al., 2004). This species belongs to the *Rosaceae* family and is a hybrid between *P. glabra* and *P. serrulata*. *P*. × *fraseri* has been used as an ornamental plant due to its bright flower-like leaves and ability to adjust to disadvantageous environmental conditions, such as cold temperatures, drought, and poor soil; the economic and ecological value of the species is increasingly receiving attention (Deng et al., 2004). *Eugenia uniflora* ‘Etna Fire’ is a new variety that is rapidly spreading in the Mediterranean environments as ornamental shrub. Preliminary studies on drought stress tolerance revealed that *Eugenia uniflora* rapidly reacts to drought stress and does not show visible stress symptoms for more than 1 month in limited water availability.

Therefore, the aim of our study was to understand the physiological and biochemical mechanisms involved in drought adaptation, especially their correlations, in a fast adaptation species, such as *Eugenia uniflora* ‘Etna Fire,’ and in a species with slower reaction to water stress, such as *P.* × *fraseri* ‘Red Robin.’ Physiological and biochemical parameters were measured such as gas exchanges, chlorophyll *a* fluorescence, RWC, chlorophyll content, proline, lipid peroxidation, and antioxidant enzyme activities (SOD, CAT, and GPX). We hypothesize that antioxidant enzyme activities contribute to different drought tolerance levels in two analyzed species. A second hypothesis is that there would be positive correlations among photosynthetic performance, proline and MDA accumulation. The third hypothesis is that there would be a correlation between photosynthetic performance and level of antioxidant enzymes in both species.

## Materials and Methods

### Plant Materials and Treatments

The experimental trial was carried out in an unheated greenhouse located near Catania, Italy (37°41′N 15°11′E 89 m a.s.l.).

Three months old rooted cuttings of *Eugenia uniflora* L. ‘Etna Fire’ and *P. × fraseri* Dress ‘Red Robin’ were transplanted into 3 L plastic pots (16 cm) filled with peat and perlite (2:1 v/v) amended with 2 g L^-1^ of Osmocote Plus (14:13:13 N, P, K plus microelements). The plants were daily watered to container capacity during 2 weeks prior starting the treatments. Plants were grouped into three replicates (ten plants per replicate) and were submitted for 30 days (May–June 2014) to three treatments: well-watered (WW), moderate drought stress (MD), and severe drought stress (SD). Which one received, every 2 days, around 100% (WW), 75% (MD), and 35% (SD) of water considering the evapotranspiration rate performed using the gravimetric method. The water added to each pot during the experimental period was 2.39, 1.67, and 0.84 L for WW, MD, and SD, respectively for plants of *Eugenia uniflora* and 1.96, 1.37, and 0.69 L for plants of *P. × fraseri*. The electrical conductivity of water used was 0.9 dS m^-1^.

### Gas Exchange Measurements

On days 7, 14, 21, 28 of the experimental period, the gas exchange was measured between 10:00 and 13:00 (solar time). Net photosynthetic rate (*A*), stomatal conductance (*G*s), and transpiration rate (*E*) were measured on mature and fully expanded leaves using a CO_2_/H_2_O IRGA (LCi, ADC Bioscientific Ltd., Hoddesdon, UK). All the photosynthesis measurements were performed on the outer fully expanded leaves sampled on branches located in the middle of the canopy.

### Chlorophyll a Fluorescence

The maximum quantum efficiency of PSII photochemistry was measured on days 7, 14, 21, 28 of the experimental period, using a modulated chlorophyll fluorometer OS1-FL (Opti-Sciences Corporation, Tyngsboro, MA, USA). Leaves were dark-adapted for 30 min prior to measurements. Fv/Fm ratio was calculated using the formula (Fm-F0)/Fm, where Fm is maximal fluorescence yield of the dark-adapted state and F0 is minimum fluorescence yield (Maxwell and Johnson, 2000).

### Relative Water Content

The RWC was measured on days 7, 14, 21, and 28 of the experimental period between 12:00 and 14:00 (solar time, midday) on fully opened leaves. Five leaf disks of 10 mm in diameter were excised from the interveinal areas of each plant. For each replicate, 30 disks were pooled, and their fresh weights (FW) were determined. They were floated on distilled water in Petri dishes for 4 h to regain turgidity and then the turgid tissue was quickly blotted to remove excess water and reweighed [turgid weight (TW)]. The samples were dried at 80°C for 24 h to determine the dry weights (DW; Rouphael et al., 2008). The RWC was calculated using the formula:

RWC% =(FW−DW/TW−DW)*100.

### Determination of Chlorophyll Content

At the end of the trial chlorophyll a (Chl_a_) and chlorophyll b (Chl_b_) were analyzed according to Yang et al. (1998). For the extraction 250 mg fresh material of three leaves per replication were collected and dried with liquid nitrogen and ground it into powder with pestle and mortar; after ground were extract total pigments with 5mL of 80% acetone. Next, the crude extract was centrifuge at *1500 g* for 5 min. The supernatant was kept and discarded the pellet. Quantification was performed by spectrophotometry at 663.6 and 646.6 nm. The calculation of chlorophylls were done through the following formulas (Porra et al., 1989): Chl_a_ = (12.25 × Abs_663.6_) – (2.55 × Abs_646.6_) (μg/mL); Chl_b_ = (20.31 × Abs_646.6_) – (4.91 × Abs_663.6_) (μg/mL).

### Estimation of Proline Content

The amount of free proline (Pro) in fresh material was determined as reported by Ahmad et al. (2008) with slight adjustments. Fresh material (1 g) was homogenized in 5 ml of 3% aqueous sulfosalicylic acid. The homogenate was centrifuged at *14000 g* for 15 min. A 2 mL aliquot of the supernatant was mixed with an equal volume of acetic acid and acid ninhydrin and incubated for 1 h at 100°C. The reaction was terminated in an ice bath and extracted with 4 mL of toluene. The extract was vortexed for 20 s. The absorbance was determined spectrophotometerically at 525 nm using toluene for a blank, L-proline as the standard.

### Estimation of Lipid Peroxidation

Malondialdehyde content was measured as reported by Li et al. (2010). The small pieces of leaves (approximately 0.5 g) were homogenized in 1.5 mL of 5% trichloroacetic acid (weight/volume). The homogenate was centrifuged at *5000 g* for 10 min, and then the supernatant was diluted to 10 mL. The diluted extract (2 mL) was mixed with the same volume of 0.67% TBA. The mixture was incubated in boiling water (95–100°C) for 30 min, and then centrifuged at *5000 g* for 10 min. MDA content in the aqueous phase was calculated based on the following formula: C (μmol/L) = 6.45 × (A532 - A600) - 0.56 × A450.

### Extraction and Assay of Antioxidant Enzymes

For enzyme extraction, 0.5 g leaf powder were extracted with 4 mL of extraction buffer (50 mM potassium phosphate, 1 mM ethylenediaminetetraacetic acid [EDTA], 1% polyvinylpyrrolidone [PVP], 1 mM dithiothreitol [DTT], and 1 mM phenylmethylsulfonyl [PMSF], pH 7.8). The extractions were centrifuged at *15000 g* for 30 min at 4°C (Bian and Jiang, 2009). The supernatant was collected and stored at -80°C for SOD, CAT, and GPX. The protein content was determined using Bradford’s method (1976). The SOD (SOD; EC 1.15.1.1) activity was assayed by monitoring the inhibition of photochemical reduction of nitro blue tetrazolium (NBT) according to the method of Giannopolitis and Ries (1977). One unit of SOD activity was defined as the amount of enzyme required to cause 50% inhibition of the reduction of NBT as monitored at 560 nm. SOD activity was expressed as units mg^-1^ protein. The CAT (CAT; EC 1.11.1.6) was analyzed according to Aebi (1984); briefly 10–40 μL extract was added to 810–840 μL potassium phosphate buffer (50 mM, pH 7). The reaction was started by the addition of 150 μL of H_2_O_2_ solution in phosphate buffer and followed by monitoring the decrease in absorbance at 240 nm at 20°C for 1–2 min (this resulted in a decrease in absorbance of approximately 0.05 U; Aguilera et al., 2002). CAT activity was expressed as μmol min^-1^ mg^-1^ protein. The GPX (GPX; EC 1.11.1.7) activity was measured using the method described by Ruley et al. (2004). In the presence of H_2_O_2_ GPX catalyzes the transformation of guaiacol to tetraguaiacol (brown product). A reaction mixture consisting of the suitable quantity of enzymatic extracts, with equal amount of 17 mM H_2_O_2_ and 2% guaiacol to get the final volume of 1 mL. The increase in absorbance was then assayed for 3 min at 510 nm. Activity was measured as appearance of tetra-guaiacol. One unit of enzyme activity was defined as an increase in absorbance of 0.001 min^-1^ at 510 nm. GPX activity was expressed as μmol min^-1^ mg^-1^ protein.

### Climate Conditions

The mean air temperature and relative humidity during the experimental periods were recorded on a data logger (CR 1000; Campbell Scientific, Ltd., Loughborough, UK). The maximum and minimum temperatures were 17.6°C and 23.0°C, respectively, and the mean relative humidity levels ranged from 63 to 97%.

### Statistical Analysis

The experiment was randomized complete block design with three replicates of 10 plants. The data were subjected to analysis of variance (ANOVA) and means were compared with Tukey-test (*P* < 0.05) using CoStat release 6.311 (CoHort Software, Monterey, CA, USA). For each species, the values of each biochemical variable were compared by repeated-measure analysis of variance, with “drought intensity” as between-subject effects and “drought time” as within-subject effects. Pearson’s correlation coefficient (r) was calculated among all the physiological parameters and between the biochemical parameters.

## Results

### Gas Exchange Analyses

**FIGURE 1:**
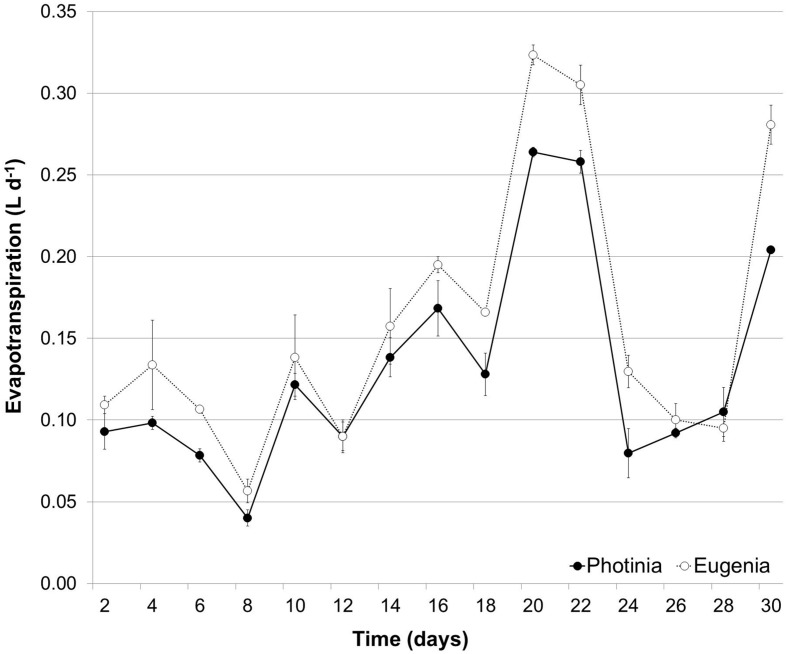
**Temporal variation of Evapotranspiration.** Evapotranspiration during the experimental period in *Eugenia uniflora* (∘) and *Photinia Photinia × fraseri* (•) subjected to well-watered (WW) treatment. Values represent mean ± E.S. (*n* = 3).

During the trial period the evapotranspiration rate did not show significant differences between the two species; the amount of water lost by evapotranspiration in WW plants was 0.159 L d^-1^ for *Eugenia uniflora* and 0.131 L d^-1^ for *P. × fraseri* (**Figure 1**).

The net photosynthesis, stomatal conductance and transpiration rate showed similar trends; differences were observed for stomatal conductance between the two species. In *Eugenia uniflora* the decrease of net assimilation was significant soon after the first week of stress and remained significant for the entire experimental period. Under control conditions, net photosynthesis was about 8 μmol CO_2_ m^-2^ s^-1^. MD stress induced a progressive decrease in net photosynthesis with values from 4.9 μmol CO_2_ m^-2^ s^-1^ at 7^th^ day to 3.0 μmol CO_2_ m^-2^ s^-1^ at 28^th^ day. Drastic reductions were observed in SD with values from 2.1 μmol CO_2_ m^-2^ s^-1^ at 7^th^ day to 0.7 μmol CO_2_ m^-2^ s^-1^ at 28^th^ day (**Table 1**). In *P. × fraseri* net photosynthesis decreased after the second week of treatments. In plants WW, net photosynthesis was about 10 μmol CO_2_ m^-2^ s^-1^. MD stress induced a progressive decrease in net photosynthesis with values ranging from 8.3 μmol CO_2_ m^-2^ s^-1^ at 14^th^ day to 3.9 μmol CO_2_ m^-2^ s^-1^ at 28^th^ day. SD showed the lowest values from 3.3 μmol CO_2_ m^-2^ s^-1^ at 14^th^ day to 1.1 μmol CO_2_ m^-2^ s^-1^ at 28^th^ day (**Table 1**). In both genotypes the photosynthesis reduction was related to stomatal closure; there was, in fact, a significant correlation between the two parameters.

**Table 1:** Effects of different treatments on net photosynthesis, stomatal conductance, and transpiration rate in *Eugenia uniflora* ‘Etna fire’ and *P. × fraseri* ‘Red Robin’ species on days 7, 14, 21, and 28 in WW, MD, and SD treatments.

Species	Treatment	A (μmol CO_2_ m^2^ s^-1^)				Gs (mmol m^-2^ s^-1^)				E (mmol m^-2^ s^-1^)			
				
	Days	7	14	21	28	7	14	21	28	7	14	21	28
*Eugenia*	WW	8.0 ± 0.9a	8.3 ± 0.4a	8.5 ± 0.4a	7.9 ± 0.2a	87.5 ± 6.9a	108.5 ± 11.5a	101.5 ± 4.1a	119.0 ± 10.8a	2.2 ± 0.2a	2.8 ± 0.5a	3.4 ± 0.2a	4.7 ± 0.3a
	MD	4.9 ± 0.3b	5.4 ± 0.3b	3.9 ± 0.4b	3.0 ± 0.1b	20.0 ± 3.7b	40.0 ± 4.5b	25.0 ± 2.2b	23.3 ± 4.2b	0.4 ± 0.1b	1.1 ± 0.0b	0.9 ± 0.1b	0.9 ± 0.1b
	SD	2.1 ± 0.2c	1.8 ± 0.1c	0.9 ± 0.3c	0.7 ± 0.2c	20.3 ± 2.6b	15.0 ± 2.2c	17.5 ± 2.1b	17.5 ± 1.5b	0.3 ± 0.1b	0.3 ± 0.1b	0.2 ± 0.0c	0.6 ± 0.1b
	*P*	***	***	***	***	***	***	***	**	***	**	***	***
*Photinia*	WW	12.0 ± 0.6	11.3 ± 0.6a	10.9 ± 0.5a	7.4 ± 0.5a	131.8 ± 9.8	133.0 ± 21.7a	154.0 ± 16.5a	113.2 ± 19.1a	2.8 ± 0.3	3.6 ± 0.1a	4.1 ± 0.2a	3.9 ± 0.2a
	MD	11.2 ± 0.7	8.3 ± 0.3b	3.4 ± 0.2b	3.9 ± 0.2b	118.3 ± 26.5	55.0 ± 6.2b	34.3 ± 5.7b	25.7 ± 2.2b	2.2 ± 0.4	1.2 ± 0.1	1.1 ± 0.2b	1.0 ± 0.1b
	SD	11.2 ± 0.6	3.3 ± 0.1c	2.0 ± 0.3c	1.1 ± 0.3c	108.3 ± 23.7	21.7 ± 1.8b	18.3 ± 4.4b	12.0 ± 2.4b	2.1 ± 0.5	0.6 ± 0.0c	0.6 ± 0.1b	0.3 ± 0.0c
	*P*	n.s.	***	***	***	n.s.	***	***	***	n.s.	***	***	***


*Values represent mean ± E.S. of three replications per treatment. Different letters indicate significant differences (*P* < 0.05) with respect to irrigation treatment for each species. n.s., not significant; ^∗∗^*P* < 0.01, ^∗∗∗^*P* < 0.001 represent significance of main effects from ANOVA. The values in the same column followed by the same letter are not significantly different at *P* < 0.05 (Tukey test). A: net photosynthesis; Gs: stomatal conductance; E: transpiration rate.*

Significant reductions in *Gs* in *Eugenia uniflora* were induced by drought treatments (**Table 1**). On day 7 of drought stress, stomatal conductance decreased from 87.5 mmol m^-2^ s^-1^ (WW) to 20.0 and 20.03 mmol m^-2^ s^-1^ (MD and SD) respectively. The reduction has been progressive and at 28^th^ day reached values of 23.3 mmol m^-2^ s^-1^ and 17.5 mmol m^-2^ s^-1^ in MD and SD respectively. In *P. × fraseri* values of stomatal conductance significantly decreased after the second week until the end of the experimental period. MD stress induced a progressive decreasing with values from 55.0 mmol m^-2^ s^-1^ at 14^th^ day to 25.7 mmol m^-2^ s^-1^ at 28^th^ day. The SD treatment showed drastic reductions with values ranging from 21.7 mmol m^-2^ s^-1^ at 14^th^ day to 12.0 mmol m^-2^ s^-1^ at 28 days (**Table 1**).

### Chlorophyll a Fluorescence

The maximum quantum efficiency of PSII (Fv/Fm) in both stressed species was lower than control at the end of the trial (**Table 2**). Minimal fluorescence (F0) was higher in stressed plants but statistical difference were not found in all sampling points. At beginning of the experiment, the *Eugenia uniflora* plants had lower Fv/Fm ratio compared the *P. × fraseri* plants. *Eugenia uniflora* plants appeared the most stressed. In water deficit (MD and SD) treatments, the value of Fv/Fm reached, in fact, values respectively of 0.69 and 0.67. In *P. × fraseri* plants, however, there was a decrease of this parameter but only in SD where reached the mean value of 0.73 (**Table 2**).

**Table 2:** Effects of different treatments on relative water content, minimum fluorescence yield, and maximum quantum efficiency of PSII in *Eugenia uniflora* ‘Etna fire’ and *Photinia × fraseri* ‘Red Robin’ species on days 7, 14, 21, and 28 in WW, MD, and SD treatments.

Species	Treatment	RWC (%)				F0				Fv/Fm			
				
	Days	7	14	21	28	7	14	21	28	7	14	21	28
*Eugenia*	WW	83.7 ± 0.9	83.8 ± 2.6	81.3 ± 1.7	82.9 ± 1.6a	241.0 ± 7.8	222.3 ± 27.7	182.3 ± 7.9b	212.7 ± 13.7	0.79 ± 0.01	0.78 ± 0.01	0.77 ± 0.02a	0.76 ± 0.02a
	MD	83.9 ± 1.4	82.3 ± 3.5	80.1 ± 0.7	83.2 ± 1.1a	223.3 ± 21.7	174.3 ± 10.1	162.0 ± 5.0b	224.3 ± 31.5	0.78 ± 0.01	0.76 ± 0.01	0.76 ± 0.00a	0.69 ± 0.00b
	SD	77.9 ± 1.8	77.1 ± 2.3	75.8 ± 2.5	73.3 ± 2.3b	214.0 ± 27.9	162.0 ± 6.1	229.0 ± 16.0a	223.3 ± 10.7	0.78 ± 0.01	0.78 ± 0.01	0.66 ± 0.03b	0.67 ± 0.01b
	*P*	n.s.	n.s.	n.s.	*	n.s.	n.s.	*	n.s.	n.s.	n.s.	*	*
*Photinia*	WW	68.9 ± 3.4	68.9 ± 1.5a	73.1 ± 1.0a	77.9 ± 1.4a	203.7 ± 9.2	136.0 ± 4.9b	148.7 ± 2.2b	173.7 ± 13.7	0.82 ± 0.00	0.81 ± 0.01a	0.79 ± 0.01	0.79 ± 0.00a
	MD	65.6 ± 2.6	66.2 ± 0.8a	66.4 ± 0.9b	67.3 ± 0.4b	194.3 ± 3.3	163.3 ± 5.6a	182.0 ± 10.4ab	163.7 ± 4.7	0.84 ± 0.00	0.76 ± 0.01b	0.76 ± 0.02	0.78 ± 0.00a
	SD	70.1 ± 1.3	56.6 ± 1.1b	57.4 ± 0.5c	55.7 ± 3.9c	190.7 ± 4.7	155.3 ± 6.7ab	175.3 ± 5.2a	200.3 ± 19.6	0.79 ± 0.05	0.76 ± 0.01b	0.76 ± 0.01	0.73 ± 0.01b
	*P*	n.s.	***	***	***	n.s.	*	*	n.s.	n.s.	**	n.s.	***


*Values represent mean ± E.S. of three replications per treatment. Different letters indicate significant differences (*P* < 0.05) with respect to irrigation treatment for each species. n.s., not significant; ^∗^*P* < 0.05, ^∗∗^*P* < 0.01, ^∗∗∗^*P* < 0.001 represent significance of main effects from ANOVA. The values in the same column followed by the same letter are not significantly different at *P* < 0.05 (Tukey test). RWC: relative water content; F0: minimum fluorescence yield; Fv/Fm: maximum quantum efficiency of PSII.*

### Relative Water Content

In *Eugenia uniflora* the RWC appeared to be influenced by water treatments only at the end of trial and, in the thesis more stressed (SD), the decrease from the control was of 12%; in *P. × fraseri*, however, the decrease was significant at the second week for SD and in the third one for MD (**Table 2**).

### Chlorophyll Content

**FIGURE 2:**
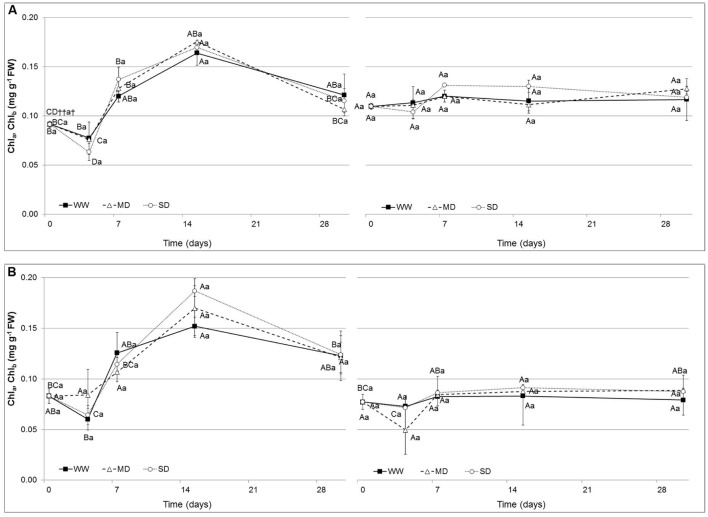
**Chlorophyll a (Chl_a_) and Chlorophyll b (Chl_b_) (mg g^-1^ FW) in the leaves of *Eugenia uniflora***(A)** and *P. × fraseri***(B)** at 0, 4, 7, 15 and 30 days.** (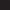) Well-watered (WW) treatment; (Δ) Moderate-drought (MD) stressed; (∘) Severe-drought (SD) stressed. Values represent mean ± E.S. (*n* = 3). Different letters indicate significant difference among treatments (†) and days for treatment (††) at *P* < 0.05 according to Tukey test. Values represented by the same upper case letters, between time of treatment and same lower case letters, among treatments are not significantly different by Tukey test.

The chlorophyll content (Chl_a_ and Chl_b_; **Figure 2**) did not show significant changes in both species. Values ranged from 0.09 to 0.16 mg g^-1^ FW in WW, from 0.09 to 0.18 mg g^-1^ FW in MD and from 0.09 to 0.17 mg g^-1^ FW in SD for Chl_a_, while from 0.11 to 0.12 mg g^-1^ FW in WW, from 0.11 to 0.13 mg g^-1^ FW in MD and from 0.11 to 0.13 mg g^-1^ FW in SD for Chl_b_ in *Eugenia uniflora*. In *P. × fraseri* plants, values were comprised from 0.08 to 0.15 mg g^-1^ FW in WW treatment, from 0.09 to 0.7 mg g^-1^ FW in MD and from 0.09 to 0.18 mg g^-1^ FW in the SD treatment for Chl_a_, while for Chl_b_ values were comprised from 0.08 to 0.08 mg g^-1^ FW in the WW treatment, from 0.08 to 0.09 mg g^-1^ FW in MD and from 0.08 to 0.09 in the SD treatment.

### Proline Content

**FIGURE 3:**
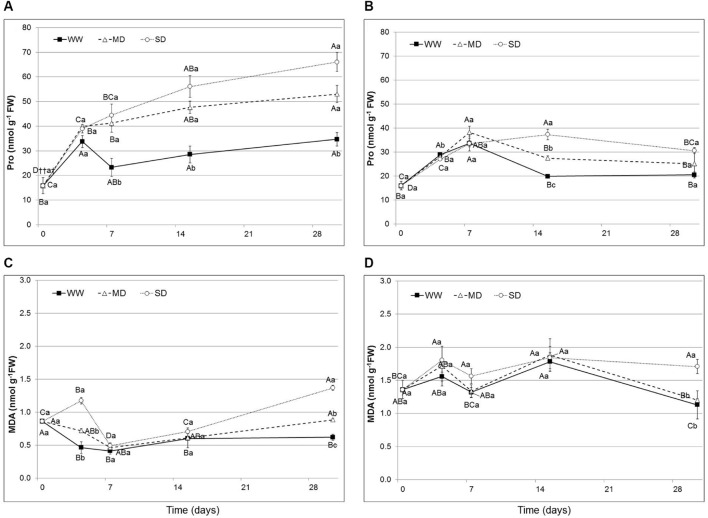
**Proline (Pro) and Malondialdehyde (MDA) content in the leaves of *Eugenia uniflora***(A–C)** and *P. × fraseri***(B–D)** at 0, 4, 7, 15 and 30 days.** (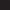) WW treatment; (Δ) MD stressed; (∘) SD stressed. Values represent mean ± E.S. (*n* = 3). Different letters indicate significant difference among treatments (†) and days for treatment (††) at *P* < 0.05 according to Tukey test. Values represented by the same upper case letters, between time of treatment and same lower case letters, among treatments are not significantly different by Tukey test.

The amount of leaf proline content increased with the exposure time to the water stress. In *Eugenia uniflora* as early as the 7^th^ day a significant difference in the content of proline was observed. The content of this amino acid reached its maximum value after 30 days, when it was 53.04 nmol g^-1^ FW in the thesis MD and 66.08 nmol g^-1^ FW in the thesis SD (**Figure 3A**). In *P. × fraseri*, however, significant differences were observed from the day 15, with a content to 25.04 and 30.57 nmol g^-1^ FW respectively in MD and SD (**Figure 3B**).

### MDA Content

Lipid peroxidation was determined by evaluating the MDA contents in leaf tissues. In our study, the MDA significantly increased only after 30 days of stress in both species. In *Eugenia uniflora*, there was an increase of 43% (0.88 nmol g^-1^ FW) and 55% (1.37 nmol g^-1^ FW) respectively in the MD and SD treatments (**Figure 3C**), while in *P. × fraseri* the increase was only significant for the most stressed treatment after 30 days, with an increase of 34% (1.71 nmol g^-1^ FW) in SD (**Figure 3D**).

### Activity of Antioxidant Enzymes

**FIGURE 4:**
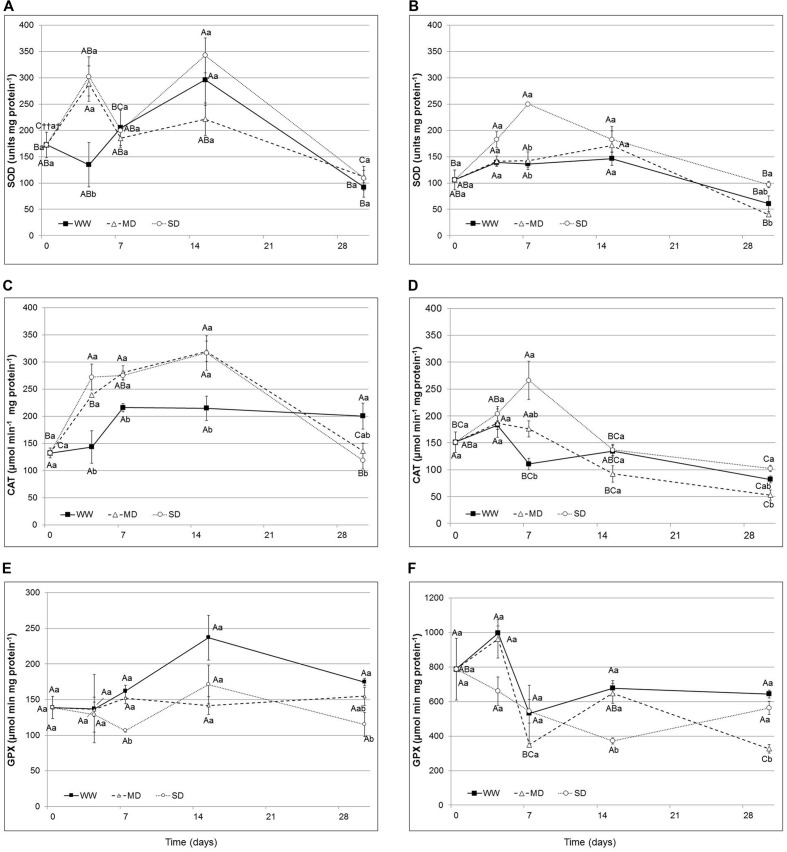
**Superoxide dismutase (SOD), Catalase (CAT), and Peroxidase (GPX) activity in the leaves of *Eugenia uniflora***(A–C,E)** and *P. × fraseri***(B–D,F)** at 0, 4, 7, 15 and 30 days.** (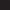) WW treatment; (Δ) MD stressed; (∘) SD stressed Values represent mean ± E.S. (*n* = 3). Different letters indicate significant difference among treatments (†) and days for treatment (††) at *P* < 0.05 according to Tukey test. Values represented by the same upper case letters, between time of treatment and same lower case letters, between treatments are not significantly different by Tukey test.

The activities of antioxidant enzymes, SOD, CAT and GPX, showed different behavior to water stress and results are shown in **Figure 4** (from A to F). SOD activity showed in *Eugenia uniflora* a significant increase (55 and 53% respectively in MD and SD) after 4 days of stress (**Figure 4A**). In *P. × fraseri*, SOD activity was significantly affected in the SD treatment, at 7^th^ day, and showed an increase by 46% (**Figure 4B**). At the end of the experimental period, stressed plants reduced the SOD activity, in fact in both species there were not differences (**Figures 4A,B**).

The CAT activity increased in *Eugenia uniflora* exposed to drought stress during the experimental period; only at 30^th^ day the values were similar to the control (**Figure 4C**). In *P. × fraseri*, the levels of CAT activity remained similar for all treatments and during the entire period of stress; there was an increase only at the 7^th^ day for both levels of water stress (**Figure 4D**).

The GPX activity in *Eugenia uniflora* showed a significant, but transient increase at 7^th^ day (by 34% in WW and 30% in MD) then the activity returned to original level after 30 days (**Figure 4E**). In *P. × fraseri*, instead, the GPX activity values were higher than *Eugenia uniflora* in all treatments, with significant differences at the end of experimental period with an increase by 47 and 42% in WW and MD at 7^th^ day and by 49 and 42% in WW and SD at 30^th^ day (**Figure 4F**).

### Correlation Analysis

Correlation coefficients among water stress indexes analyzed by Pearson’s correlation are listed in **Tables 3** and **4**. Water stress indexes related to several parameters showed significant correlations. In both species the water stress scores displayed positive correlations with the all physiological parameters, while the proline showed a negative correlation with the all physiological parameters (**Tables 3** and **4**). In *Eugenia uniflora* the catalase activity showed a negative correlation with the gas exchanges while the GPX activity showed a positive correlation (**Table 3**). In *P. × fraseri* GPX activity was correlated positively with all physiological parameters except with RWC (**Table 4**).

**Table 3:** Pearson’s correlation coefficients among physiological and biochemical parameters from *Eugenia uniflora* ‘Etna fire’ exposed to water stress.

	A	Gs	E	F_V_/F_m_	RWC	CAT	GPX	MDA	SOD	PRO	Chl_a_	Chl_b_
A	–											
Gs	0.954***	-										
E	0.873***	0.945***	–									
F_v_/F_m_	0.548***	0.456**	0.375*	–								
RWC	0.574***	0.431**	0.358*	0.346*	–							
CAT	-0.322*	-0.382*	-0.357*	0.056	-0.143	–						
GPX	0.390*	0.371*	0.441**	0.058	0.211	0.140	–					
MDA	0.180	0.283	0.462**	-0.101	-0.070	-0.160	0.063	–				
SOD	-0.155	-0.251	-0.2987	0.036	-0.092	0.578***	0.392*	-0.328*	–			
PRO	-0.800***	-0.732***	-0.645***	-0.684***	-0.522***	0.185	-0.247	0.043	-0.067	–		
Chl_a_	-0.087	-0.079	-0.010	-0.363*	-0.206	0.451**	0.250	-0.107	0.130	0.235	–	
Chl_b_	-0.422**	-0.375*	-0.250	-0.368*	-0.322*	0.017	-0.138	0.020	-0.042	0.476**	0.225	–


*Each square indicates the Pearson’s correlation coefficient of a pair of parameters. A: net photosynthesis (μmol CO_*2*_ m^*2*^ s^-*1*^), Gs: stomatal conductance (mmol m^-*2*^ s^-*1*^), E: traspiration rate (mmol m^-*2*^ s^-*1*^), Fv/Fm: cholorophyll fluorescence, RWC: relative water content (%), CAT: catalase (μmol min^-*1*^ mg protein^-*1*^), GPX: peroxidase (μmol min^-*1*^ mg protein^-*1*^), MDA: malondyaldeide (nmol g^-*1*^ FW), SOD: superoxide dismutase (units mg protein^-*1*^), Pro: proline (nmol g^-*1*^ FW), Chla: chlorophyll a (mg g^-*1*^ FW), Chlb: chlorophyll b (mg g^-*1*^ FW). Correlation was significant at the ^∗^*P* < 0.05; ^∗∗^*P* < 0.01; ^∗∗∗^*P* < 0.001.*

**Table 4:** Pearson’s correlation coefficients among physiological and biochemical parameters from *P. × fraseri* ‘Red Robin’ exposed to water stress.

	A	Gs	E	F_V_/F_m_	RWC	CAT	GPX	MDA	SOD	PRO	Chl_a_	Chl_b_
A	–											
Gs	0.927***	–										
E	0.854***	0.931***	–									
F_v_/F_m_	0.700***	0.636***	0.579***	–								
RWC	0.597***	0.618***	0.746***	0.391*	–							
CAT	0.148	0.059	-0.102	0.049	-0.253	–						
GPX	0.512***	0.483**	0.357*	0.457***	0.250	0.279	–					
MDA	-0.359*	-0.173	-0.166	-0.296	0.023	-0.181	-0.081	–				
SOD	-0.109	-0.138	-0.263	-0.221	-0.363*	0.715**	0.104	-0.025	–			
PRO	-0.363*	-0.467***	-0.543***	-0.364*	-0.478**	0.281	-0.375*	-0.143	0.376*	–		
Chl_a_	-0.447**	-0.288	-0.183	-0.338*	-0.174	-0.325*	-0.445**	0.290	0.061	0.217	–	
Chl_b_	-0.330*	-0.216	-0.199	-0.419**	-0.131	-0.158	-0.381*	0.200	-0.015	0.214	0.398	–


*Each square indicates the Pearson’s correlation coefficient of a pair of parameters. A: net photosynthesis (μmol CO_*2*_ m^*2*^ s^-*1*^), Gs: stomatal conductance (mmol m^-*2*^ s^-*1*^), E: traspiration rate (mmol m^-*2*^ s^-*1*^), Fv/Fm, cholorophyll fluorescence, RWC: relative water content (%), CAT: catalase (μmol min^-*1*^ mg protein^-*1*^), GPX: peroxidase (μmol min^-*1*^ mg protein^-*1*^), MDA: malondyaldeide (nmol g^-*1*^ FW), SOD: superoxide dismutase (units mg protein^-*1*^), Pro: proline (nmol g^-*1*^ FW), Chl_*a*_: chlorophyll a (mg g^-*1*^ FW), Chl_*b*_: chlorophyll b (mg g^-*1*^ FW). Correlation was significant at the ^∗^*P* < 0.05; ^∗∗^*P* < 0.01; ^∗∗∗^*P* < 0.001.*

## Discussion

Drought stress induces different plant responses, which include physiological and metabolic changes (Tian and Lei, 2007). Plant response to drought stress is affected by climatic, edaphic, and agronomic factors (Anjum et al., 2011). The plant susceptibility to drought stress varies in dependence of stress degree, different interactions among other stress factors, plant species and their developmental stages (Demirevska et al., 2009).

Our results showed a different response to the stress of the two species, related to their different tolerance degree.

Relative water content was higher in *Eugenia uniflora* plants compared to *P. × fraseri*, demonstrating the ability of this species to retain water. Leaf RWC is considered a reliable indicator that reflects the water content in relation to maximum water content. Therefore, it indicates the level of hydration (Rosales-Serna et al., 2004). It has been demonstrated that water deficit diminishes RWC in several species of plants, including chives, wheat and turfgrass (DaCosta and Huang, 2007). Generally high values of RWC are considered as index of stress tolerance (Tounekti et al., 2011), as demonstrated by Larbi and Mekliche (2004) on wheat varieties sensitive or resistant to dry. RWC is also considered a good indicator of water stress severity. In several Mediterranean shrubs, RWC values around 80% are considered as good water availability (Munné-Bosch et al., 2003). In other species, such as *Arbutus unedo* RWC values of 68% are considered as moderate water stress conditions, while values of 50% are considered very stressful conditions (Munné-Bosch and Peñuelas, 2004). On the basis of this classification, the *Photinia* plants in the SD treatments are considered under severe drought stress.

Both species with increasing of water stress have shown a reduction in the assimilation process. The significant decrease of *Gs* in drought treatments of both species suggests an efficient adaptive transpiration control (Hessini et al., 2008). Many studies have shown the decreased photosynthetic activity under drought stress due to stomatal or non-stomatal mechanisms (Samarah et al., 2009). In drought tolerant species, the reduction of photosynthesis is due to stomatal closure and limitation of water losses. In the drought sensitive plants, the reduction of net photosynthesis is mainly due to water shortage and plants undergo severe damages. Our results have shown, in both species, a positive correlation between photosynthesis and stomatal conductance, in order to prove that stomatal closure increases with the increase of drought stress (Anjum et al., 2011). In the most part of woody species the increase of water use efficiency is connected with CO_2_ assimilation, which remained proportionally higher than water vapor loss from the stomata as an additional drought acclimation (Álvarez et al., 2011). Chlorophyll *a* fluorescence is a fast and non-destructive method for evaluating abiotic stress response in plants (Maxwell and Johnson, 2000; Pellegrini et al., 2011). The chlorophyll *a* fluorescence parameters have been used for selecting water stress tolerant plants (Percival and Sheriffs, 2002) for ornamental purpose.

The only variation of photosynthetic efficiency of PSII at the end of trial explains that the increasing of photosynthetic activity was before related to stomatal regulation in plants (Starman and Lombardini, 2006). Level of drought stress did not compromise photosynthetic efficiency (Bian and Jiang, 2009). Chlorophyll is one of the major chloroplast components for photosynthesis, and relative chlorophyll content has a positive relationship with photosynthetic rate (Guo and Li, 1996). Flexas and Medrano (2002) reported that water stress reduces green leaf color in C3 plants due to chlorophyll degradation. However, our study means that water deficits did not significantly affect the relative chlorophyll concentrations in the leaves. Lack of detectable change in chlorophyll concentrations may be due to the relatively short duration of the experiment (Toscano et al., 2014). Other authors demonstrated that the leaf chlorophyll concentrations of *Carrizo citrange* plants were not affected by relatively short-term salinity or drought-stress treatments (Pérez-Pérez et al., 2007).

Plants exposed to water and salt stress accumulate compatible solutes, as proline, for increasing the cell osmotic potential, facilitating water absorption (Ashraf and Foolad, 2007) and reducing cell injury (Anjum et al., 2011). Proline accumulation, considered a general marker of the drought tolerance (Ahmed et al., 2009; Liu et al., 2011), permits osmotic adjustment, which results in water retention and avoidance of cell dehydration (Blum, 2005). In our research, in stressed plants proline concentration was significantly higher than control plants, especially in *Eugenia uniflora*. Accumulation of proline under stress in many plant species has been correlated with stress tolerance, and its concentration has been shown to be generally higher in stress-tolerant than in stress-sensitive plants (Anjum et al., 2011). As shown by Ahmed et al. (2009) in plants of olive cultivars and as confirmed in our study, the close correlation between *A* versus *RWC* and *Pro* versus *RWC* reinforces the involvement of proline accumulation in drought tolerance mechanisms.

Malondialdehyde is a final product of lipid peroxidation (Ge et al., 2006); it has been considered an indicator of oxidative damage (Meloni et al., 2003) and it is commonly considered as one of the best physiological components of drought tolerance in plants (Xu et al., 2008). Low MDA content was associated with water stress resistance (Bacelar et al., 2007) and other environmental stresses (Sairam et al., 2000).

The plants subjected to water stress undergo an excess of reducing power, due to the limitation of the assimilation of CO_2_ which causes an increase in ROS levels and accumulation of free radicals (Schwanz and Polle, 2001). Maintaining a higher level of antioxidant enzyme activities may contribute to drought induction by increasing the capacity against oxidative damage (Sharma and Dubey, 2005). According Fan et al. (2009), the species exposed to mild and/or moderate drought stress exhibited increasing activities of antioxidant enzymes (Ge et al., 2014). Analogous results have been found in the present work, we observed high constitutive activities of SOD and CAT while the activity of GPX decreased. Under mild and/or moderate drought stress, some adapted species exhibit increases in activities of antioxidant enzymes, such as SOD and GPX (Lima et al., 2002). SOD activity is the key enzyme in the active oxygen scavenger system, because it catalyzes the dismutation of superoxide free radicals into H_2_O_2_ and O_2_; GPX and CAT further convert H_2_O_2_ into H_2_O and O_2_, and the damage caused by ROS is removed from plants (Wu et al., 2012). In *Eugenia uniflora* plants drought stress induced an increase of SOD activity for 4 days, with a subsequent decline after 7 days, while in *P. × fraseri* the increases was observed for 7 days with a decline after 15 days. SOD activity is also negative correlated with MDA in *Eugenia uniflora* and these results are confirmed by Xu et al. (2013) in herbaceous plants cultivated under salt stress; in fact, SOD activity increases while MDA content decreases.

In several study CAT activity changes increase with stress duration and its trend is species depended as well as development stages and metabolic status of plants (Chaparzadeh et al., 2004). Our results, in according to Bian and Jiang (2009) in *Poa pratensis* L. subjected to drought stress, showed an increase of APX and CAT activities. CAT and SOD are main scavengers of O_2_^⋅-^; H_2_O_2_ converting these radicals to water and molecular oxygen, reducing in stressed plants the cellular damage (Reddy et al., 2004).

Peroxidase activity was strongly reduced in *Eugenia uniflora* and lower than in *P. × fraseri*. This confirms the results obtained by other authors who argue that in stress tolerant species, the activity of GPX is higher than sensitive plants where the activity is almost nothing (Peters et al., 1989), allowing the plants greater protection from oxidative stress.

Maintaining a higher level of antioxidant enzyme activities may contribute to drought induction by increasing the capacity against oxidative damage (Sharma and Dubey, 2005). The capability of antioxidant enzymes to scavenge ROS and reduce the damaging effects may be correlated with the drought resistance of plants (Anjum et al., 2011).

The correlation analyses revealed that gas exchange parameters in *Eugenia uniflora* were significant with RWC, Pro and antioxidant enzymes such as CAT and GPX. In *P. × fraseri*, instead, the gas exchange parameters were not significant with GPX enzyme activity. The Fv/Fm values in both species were significantly correlated with RWC, Pro and chlorophyll a and b content. In *Eugenia uniflora* the Fv/Fm values were also correlated with the GPX enzyme activities.

## Conclusion

The two ornamental shrubs, even if they showed different time responses, shared the same physiological and biochemical mechanisms to counteract drought stress. *Eugenia uniflora* had a fast water stress adaptation ability and this result was reached by decreasing photosynthetic activity, enhancing the stomatal control, reducing leaf water content, increasing osmolytes accumulation (such as proline), and by the activation of the SOD and CAT enzymes compared to *P. × fraseri*. Stomatal regulation was the main physiological strategy to reduce water losses, with consequently photosynthetic activity variations, as evidenced by the high degree of correlation among these ones and physiological parameters. Proline confirmed its role of osmotic regulator under drought stress, in fact, Proline content in *Eugenia uniflora* was higher than *P. × fraseri*.

## Author Contributions

ST, EF, carried out greenhouse work, laboratory analytical determination and helped with draft the manuscript. AF contributed and writing and statistical analyses. DR contributed with experimental design, coordination and writing. All authors read and approved the final version of the manuscript.

## Conflict of Interest Statement

The authors declare that the research was conducted in the absence of any commercial or financial relationships that could be construed as a potential conflict of interest.

## Abbreviations

A: net photosynthetic rate
CAT: catalase
Chl_a_: chlorophyll a
Chl_b_: chlorophyll b
E: transpiration rate
F0: minimum fluorescence yield
Fm: maximal fluorescence yield
Fv/Fm: maximum quantum efficiency of *PSII*
G_s_: stomatal conductance
GPX: peroxidase
MDA: malondialdehyde
Pro: proline
RWC: relative water content
SOD: superoxide dismutase
